# Bacteria from Infectious Particles to Cell Based Anticancer Targeted Drug Delivery Systems

**DOI:** 10.3390/pharmaceutics13121984

**Published:** 2021-11-23

**Authors:** Mounir M. Salem-Bekhit, Abdullah M. E. Youssof, Fars K. Alanazi, Fadilah Sfouq Aleanizy, Alsuwyeh Abdulaziz, Ehab I. Taha, Amro Abd Al Fattah Amara

**Affiliations:** 1 Department of Pharmaceutics, College of Pharmacy, King Saud University, Riyadh 11451, Saudi Arabia; ayoussof@ksu.edu.sa (A.M.E.Y.); afars@ksu.edu.sa (F.K.A.); Faleanizy@ksu.edu.sa (F.S.A.); alsuwayeh@hotmail.com (A.A.); eelbadawi@ksu.edu.sa (E.I.T.); 2 Department of Microbiology and Immunology, Faculty of Pharmacy, Al-Azhar University, Cairo 11884, Egypt; 3 Kayyali Chair for Pharmaceutical Industry, Department of Pharmaceutics, College of Pharmacy, King Saud University, Riyadh 11451, Saudi Arabia; 4 Protein Research Department, Genetic Engineering and Biotechnology Research Institute, City of Scientific Research and Technological Applications, Alexandria 21934, Egypt; amroamara@hotmail.com

**Keywords:** bacterial ghosts, drug delivery, protein, vaccine, therapeutics

## Abstract

Bacterial ghosts (BGs) are empty cell envelopes of nonliving evacuated bacterial cells. They are free from their cytoplasmic contents; however, they sustain their cellular 3D morphology and antigenic structures, counting on bioadhesive properties. Lately, they have been tested as an advanced drug delivery system (DDS) for different materials like DNA, peptides, or drugs, either single components or combinations. Different studies have revealed that, BG DDS were paid the greatest attention in recent years. The current review explores the impact of BGs on the field of drug delivery and drug targeting. BGs have a varied area of applications, including vaccine and tumor therapy. Moreover, the use of BGs, their synthesis, their uniqueness as a delivery system and application principles in cancer are discussed. Furthermore, the safety issues of BGs and stability aspects of using ghost bacteria as delivery systems are discussed. Future perspective efforts that must be followed for this important system to continue to grow are important and promising.

## 1. Introduction

The term drug delivery can be defined as the approach used to deliver a drug to the patient to obtain a therapeutic effect [1]. A more specific term used among many pharmacists is drug targeting, which is an approach used to deliver a drug selectively to its intended site of action, which can be an organ, a tissue or a cell. This can benefit by increasing drug activity while reducing side effects [2]. Another idea combining these two terms is targeted drug delivery, which is the delivery of a drug using methods that will increase its concentration in some parts of the body, i.e., the site of action, while avoiding others [3]. A sufficient number of active drugs, for any ideal DDS, must be absorbed and transferred to the action target site at the right time and at the subsequent input rate. Moreover, it involves good distribution with selective transport to the site of action [4]. These factors are essential when there is only a slight margin between the toxic and effective concentrations [4,5].

The needs for targeted DDSs include many rational reasons: (i) enhancing the barrier permeation, (ii) specific targeting, (iii), decreasing side effects and (iv) enhancing drug activity; these reasons are similar to traditional delivery systems [6].

## 2. Targeted Drug Delivery System

### 2.1. The Development of a Targeted DDS

Recently, new advancement on the area of drug delivery was seen and more targeted DDSs are being researched and developed. Paul Ehrlich had discussed the concept of targeted drug delivery in 1854–1915. He first considered the idea of “magic bullets” and defined them as compounds or drugs that would have a definite attraction to microorganisms causing disease [7]. The magic bullets could selectively find out these organisms and devastate them, evading other organisms and having no damaging effects on the human cells [8,9,10,11,12]. Over the last decade, drug discovery development and advance were improved by consistent changes in pharmaceutical biotechnology and general pharmacological research, which has led to the enhancement of drug delivery scope [13]. New products using drug targeting technologies are being researched, and their number is expected to increase over the years [2].

### 2.2. Advantage of DDS

Targeted drug delivery systems have tremendous advantages, including: enhanced efficacy of medications, better safety profile, the delivery of specific and calculated amounts of the drug, ensured availability of the drug in the site of action i.e., selective delivery, enhanced drug activity at these target sites, lowered amounts of the drug available to other sites and organs, lowered amounts in systemic circulation, improved therapeutic index, fewer side effects, reduced toxicity, increased drug stability, lowered number of required doses and enhanced patient compliance [13].

### 2.3. Classification and Types of Targeted DDSs

This type of drug delivery can be used for targeting drugs to specific organ, or more specifically to cells or with even further specificity to cellular organelles [13]. We can classify targeted DDSs using three different classification methods as described in Table 1: general classification, based on site of action, and based on mechanism [14]. Each of these classes can be divided into further subclasses as follows: The first classification, which classifies targeted drug delivery, in general contains active and passive targeting [15]. The second class, which is based on the site of action, can be subdivided according to the site, which can either be an organ, a therapeutic material or a cell. The third class, which is based on mechanism, includes chemical, physical and biological targeted DDSs. This class involves using an entire biological system (such erythrocyte and ghost bacteria) or part of it (such as biologically driven ligands).

### 2.4. Challenges of Targeted DDSs

The main drawback to the development of a targeted DDS is the high cost needed for development besides the long time consumed while developing such systems [7]. In addition to the high cost and the long time, other limitations that can limit the use of targeted DDS are: first, the difficulty of targeting a drug to a specific site; second, the inability to control the amount of drug reaching non-target organs while using some delivery systems; third, the need to consider factors that decrease the amount of the drug reaching non-target sites; fourth, the need to consider both biological and physicochemical aspects of the drug and the carrier in order to enhance targeting [8]; fifth, selectivity of the targeting system may be lower when administered in vivo [2]; sixth, the need for certain antibodies or ligands to target a drug selectively to the site of action; leventh, the need for energy in some drug targeting systems e.g., in active transport and in vesicles [9], and lastly, even after using targeting systems, the probability of drug hydrolysis or degradation before reaching the target site must be considered [10].

### 2.5. Biological Targeted DDSs

This type of targeting emphasizes the increasing delivery of the active moiety (i.e., the drug) to the intended target site by making use of biological interactions, which are site-specific, such as antigen–antibody interactions and ligand–receptor binding interactions. Carriers used in active targeting include antibodies and ligands [2]. This kind of targeting can be used in drug delivery to tumor cells [16]. Recently, BGs have been recognized as DDSs for many kinds of cancer cells. Moreover, the efficacy of BGs to be targeted and internalized by leukemia, melanoma, breast cancer and colorectal carcinoma cells has been shown [17,18,19,20].

## 3. Bacterial Ghosts (BGs)

### 3.1. The Concept and Production

**Figure 1:**
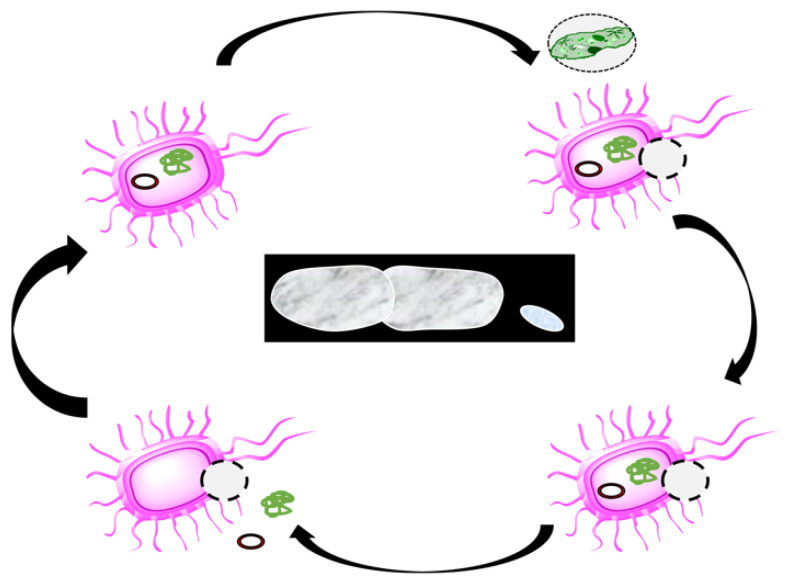
Schematic illustration of bacterial ghost preparations.

A modern delivery system for drug packing, along with vaccines, exists [21]. Generally, BGs are classically prepared via the controlled expression of PhiX174 plasmid-encoded lysis gene E. They are useful for vaccine delivery, and enhance the systemic immune response, as well as the mucosal immune system found in the gastrointestinal tract, reproductive and respiratory systems [22]. The cytoplasmic contents are ejected through the development of a transmembrane tunnel structure located in the cell wall of the used cell. The producing vacuous cell envelopes (BGs) hold minor DNA and share functional and antigenic determinants with their living counterparts, which mean that antigenic epitopes existing on the living cell are reserved on the resultant ghost [23]. BGs were prepared by different methods (Figure 1), including:

The controlled expression of lysis gene *E* of bacteriophage PhiX174 [24].

1. Sponge-Like protocol [25]. using different chemicals (Figure 1).
2. Green fluorescent protein (GFP)-dependent [26].
3. Mild high-pressure Shock [27].

### 3.2. Structure of BGs

**Figure 2:**
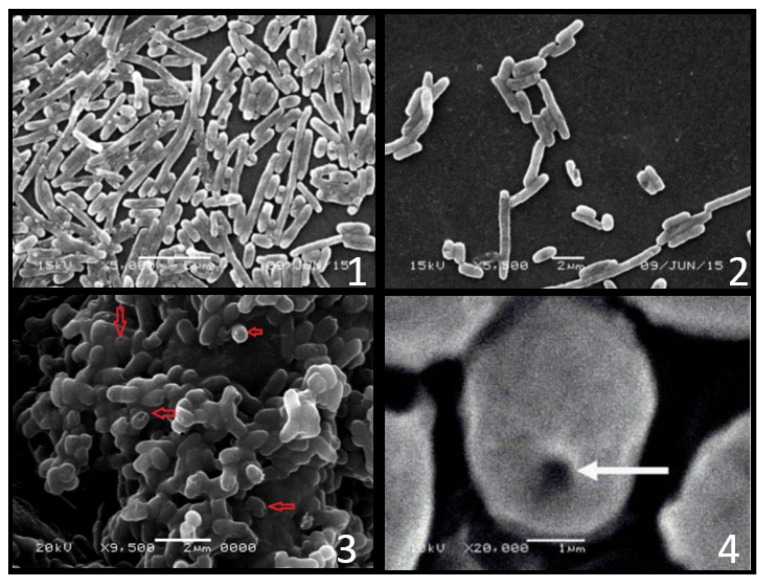
Electron microscope for the BGs at 5000× prepare by Sponge-Like protocol and sponge like reduced protocol using (**1**, **2**), tween 80 (**3**) and Lysis gene *E.* (**4**).

Based on the analytical investigations of the hydropathic regions of protein E, an E-specific lysis tunnel spanning both the inner (IM) and outer membrane (OM) was indicated. Through this lytic tunnel, the formation of the BGs was promoted by evacuating all internal content of the treated cells to the surrounding environment while keeping the periplasmic contents attached to the cell envelope [28]. The successful imaging of the E-mediated lysis tunnel in E-lysed *E. coli* was achieved using transmission electron microscopy [29]. Further scanning electron microscopy (SEM) images revealed that the formation of the transmembrane tunnel in *E. coli* resulted in a fusion of the outer and inner membrane, closing the periplasmic space (PPS). Moreover, it was reported that the lytic tunnels were located at either the center or the tip of the bacteria [30] (Figure 2).

Recently the Sponge-Like Protocol was introduced [25]. The protocol describes the use of the known concentration of some used chemical agents for microbial cells evacuation. Those compounds are SDS, NaOH, NaHCO_3_ and H_2_O_2_. To produce bacterial ghosts, the full and reduced Plackett–Burman optimization and randomization experimental design was applied to select the best physical and chemical conditions [25,31,32]. The protocol enables the evacuation of microbial cells while maintaining their 3D structure. The resulting unviable ghost cells with retained immunogenicity could be used as an immunostimulant [33,34]. Microbial ghosts prepared by this protocol can also be applied as a DDS [35]. The chemical protocol (Sponge-like) was evolved to yield cell based microbial ghosts from Gram-negative [25,31,32]. and Gram-positive [36,37] bacteria, Candida, *Saccharomyces cerevisiae* and filamentous fungi [38,39].

## 4. Application

**Figure 3:**
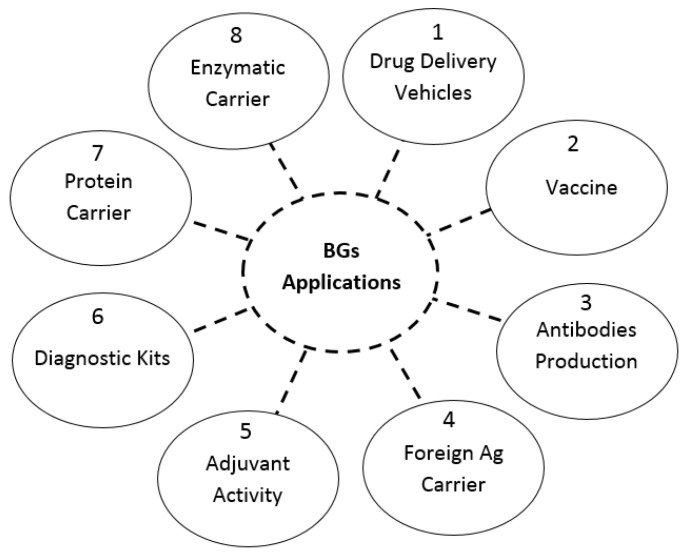
The most important clinical applications of BGs.

BGs have a wide range of biomedical applications (Figure 3). Different reports have shown that BG applicant vaccines are an extremely immunizing agent (Table 2) and, in many cases, prompt protecting immunity versus mortal challenge in animals [40]. Owing to their specific nature of being bacterial envelope developments, BGs are clever with fundamental natural adjuvant impact. They are capable of stimulating the innate and adaptive immune system without any exogenous adjuvant support (Table 3). Even though the usage of plasmid-encoded genetic material is crucial for the definitive structure of BGs, they are not classified as genetically manipulated bacteria, because they are lifeless and deficient of genetic information.

The final feature of BGs is of excessive significance for safety; there is no pathogenic island or antimicrobial resistance cassettes able to transmitted to other bacteria via gene transference. Freeze-dried preparations of BGs vaccine are stable for a long time (years) at an appropriate temperature. Similarly, it could be used as a DNA, protein carrier or as transporter and delivery vehicles for cytotoxic agents in tumor therapy [41]. As a result of the definite and specific targeting of cancer cells, BGs allow advanced treatment specificity and a reduction of the amounts of applied drugs [42]. As per enzymatic activity carrier, BGs can be applied for a novel concept of probiotics that can produce active agents from environmental substrates where they are clinically used with a specific favorite effect for the GIT [43].

i. BGs as a drug delivery system

BGs can be utilized either to target dendritic cells, macrophages [44], microvascular endothelial cells [45] and ocular surface diseases [46] or as an expression system in gene transfer to melanoma cells [47]. Applying Chang conjunctival epithelial cell line and primary cells derived from human epithelial cells of conjunctiva, BGs’ targeting potential toward these cells was studied. High degrees of BGs’ internalization into corneal cells lines, along with no cytotoxicity, were observed. Via their bioadhesive properties, BGs are able to connect with host cells and thus deliver their encapsulated payload of biomolecules or drugs at the target site [21].

ii. BGs for proteins and peptides delivery

The *E. coli* ghosts-mediated delivery of hepatitis B virus core 149 [HBcAg-149] proteins tethered to the IM or the OM of *E. coli* was examined in female BALB/c mice. Data showed that BGs succeeded in delivering HBcAg-149 to the mice used [48]. Additionally, BGs also provide a promising approach for plasmid DNA immobilizations, making them a novel carrier characterized by the intrinsic immunogenicity of the Gram-negative bacterial cell envelope [49,50]. All of these biological particles have found different applications as veterinary vaccinations and medical applications for cancer treatment and several infectious diseases.

iii. Delivery of nucleic acid via BGs

BG technology can be innovatively used in vaccine development for their response to and uptake by various types of immune cells like macrophages, monocytes and dendritic cells [51]. Therefore, nucleic acids can simply be incorporated inside a BGs’ system for gene transfer.

iv. Immunization by BGs

The immunogenic potential of *P. multocida* and *M. haemolytica* ghosts was evaluated and compared to standard marketed vaccines. Results showed that ghosts effectively protected the animal models [52]. These evoked immune responses were attributed to the presence of immunostimulating components on ghost surface, especially (Pathogen Associated Molecular Pattern) PAMPs, such as peptidoglycan, lipopolysaccharides and monophosphoryl lipid A [53].

v. As a delivery system for anticancer drugs (PK/PD)

Pathogenic bacteria can colonize a huge diversity of niches in the human body. In order for pathogenic bacteria to adhere to the milieu surface of several organs, different adherence mechanisms can be used through which bacteria can obtain access to deeper tissues by crossing the mucosal barriers and arriving at the bloodstream. This represents the principal entry portal for almost all host organs and is associated with unavoidable clinical symptoms.

**Figure 4:**
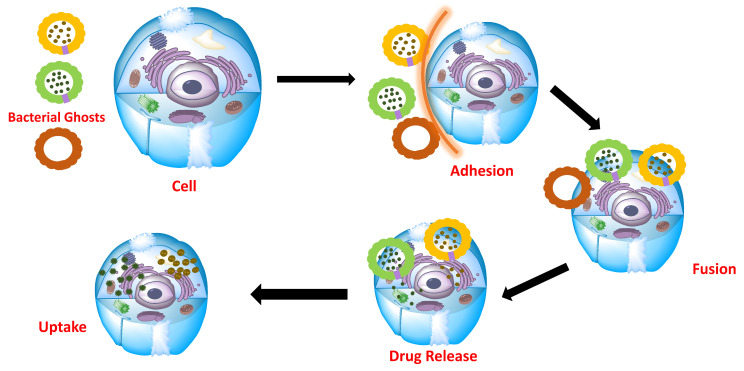
Mechanism used to deliver BGs loaded with drugs to their targets.

Host colonization through bacterial adhesion to host surfaces is a crucial stage as it protects pathogens from mechanical clearance, conferring a notable advantage towards the endogenous microbiome. Accordingly, bacteria have displayed a diversity of molecular strategies allowing them to target and attach to host cells (Figure 4). The polymeric hair-like organelles known as pili are regarded as the first class of molecular structures involved in the adherence of bacteria to host cells [54]. Via pyelonephritis-associated (P) pili at their surface, uropathogenic strains of *E. coli* (UPEC) colonize the urinary tract, resulting in kidney infections. Other UPEC strains have type I pili at their surface, which binds specifically to D-mannose receptors residing on the lining of the bladder. Additionally, another class of adhesive surface structures, type IV pili, are expressed by different Gram-negative bacteria [47].

BGs possess similar natural cellular morphology as that of the native cells as it remains protected against denaturation throughout the entire lysis process. Therefore, their natural intrinsic antigenic properties that can trigger immune responses are maintained. BGs’ antigenic elements are known as PAMPs, including lipopolysaccharides (LPS), lipid A [55] and peptidoglycan or flagella. In addition to their recognition by toll-like receptors (TLR), BGs’ PAMPs can initiate innate immune reactions as a first response as well. In experimental animals, carrying those intrinsic adjuvant properties by BGs stands for their multipurpose use in inducing both humoral and cellular immune responses. Selection of the right bacterial strain and transformation of it into ghosts contributes to treating the target organ [30].

**Table 2:** Examples of BGs as delivery system and biological carrier for recent experimental anticancer drugs.

Ghost Bacteria	Active Compound	Target Cells	Proof of Principle	Findings/Outcomes	Ref.
*Escherichia coli* NM522	DNA	Human melanoma cells	Tissue culture	BGs exhibit a high transfection efficiency; up to 82% of melanoma cells expressed the plasmid-encoded reporter gene delivered by BGs.	[17]
*Mannheimia haemolytica*	DOX	Caco-2 cells	Tissue culture	Higher antiproliferative effects of DOX on Caco-2 cells were mediated by the specific drug targeting properties of the BGs.	[56]
*E. coli*	5-FU	Caco-2 cells	Tissue culture	69.2% of the ghost-associated 5-FU was released with a significant antiproliferative effect.	[42]
*Salmonella typhimurium*	DOX	HepG2	Tissue culture	The death rate of HepG2 reached 64.5% by using of 4 μg/mL while it was about 51% using the same concentration of the free DOX. The proliferative inhibitory concentration of the DOX-loaded BG was about one third of the IC50 of the free DOX. Combined DOX showed more accumulation in early and late apoptosis than that of free DOX.	[57]
*E. coli* BL21 (DE3)	DOX	HT-29 cells	Tissue culture	DOX loaded in BG showed more apoptosis (55%) than the control and DOX solution.	[58]
*Lactobacillus acidophilus*	PG	HCT116 CRC cells	Tissue culture	PG was highly bound to LAGs cell wall with a stable bioactive entity (PG-LAGs) active against HCT116 CRC cells at the cellular and molecular levels.	[59]
*E. coli* NM522 & *M. haemolytica* A23	plasmid pEGFP-N1	SK-Mel-28 & A-375 cells	Tissue culture	High capability of cell lines to bind BGs was observed, and the Bowes cells exhibited a high expression level of GFP and the incubation of cells with plasmid-loaded BGs led up to 82% transfection efficiency.	[17]
*E. coli* Nissle1917	5-FU & zoledronic acid	4T1 tumor cells and RAW264.7 macrophages	Tissue culture & Animal studies	High loading levels of 5FU (8.8%) and ZOL (10.5%) are achieved, as well as high retention rates of bacterial viability (87%) and motion velocity (88%), leading to the accumulation of 5-FU and increases in its chemotherapeutic effect on tumors inhibition.	[22]
*E. coli* Nissle1917	Oxaliplatin	CT26 murine colon carcinoma cells (CRL-2638)	Tissue culture & Animal studies	The combination treatment has showed strong synergistic anticancer activity against the CT26 allograft, resulting in prolonged survival with complete remission in a murine model of CRC carcinomatosis.	[60]

DOX: doxorubicin; Caco-2: human colorectal adenocarcinoma cells; HepG2: human liver cancer cell line; HT-29 cells: human Caucasian colon adenocarcinoma; HCT116 CRC: colorectal cancer cells; 5-FU: 5-fluorouracil; PG: prodigiosin.

**Table 3:** Examples of BGs application as delivery system for recent experimental vaccine.

Ghost Bacteria	Active Compound	Target Cells	Proof of Principle	Finding/Outcomes	Ref.
*Helicobacter pylori*	Plain BGs	Immune cells	Oral vaccination	Coadministration of ghosts with cholera toxin as a mucosal adjuvant resulted in a complete protection of 10 of 10 and 8 of 8 mice against *H. pylori* challenge, with three animals showing sterile immunity.	[61]
*E. coli*	OmpA-HbcAg-149 Protein	Immune cells	Subcutaneous immunizations	Induced significant immune responses against HBcAg-149 in mice were observed, indicating that BGs provide an excellent carrier system for antigen delivery.	[62]
*Salmonella typhimurium*–LTB	MontanideTM ISA 70VG	Immune cells	Intramuscular immunization	Injection of *S. typhimurium*-LTB ghost with or without Montanide(TM) ISA70VG adjuvant is capable of inducing protective immunity against the virulent *S. typhimurium* infection in chickens.	[63]
*E. coli* O157:H7	staphylococcal nuclease A	Immune cells	Oral immunization	Immunized mice showed 86% protection against lethal challenge with a heterologous EHEC strain after single-dose oral immunization and 93.3% protection after one booster at day 28, whereas the controls showed 26.7% and 30% survival, respectively. These results indicate that it is possible to develop an efficacious single-dose oral EHEC BG vaccine.	[64]
*Salmonella enteritidis*	flagellin (FliC) antigen	Immune cells	Intramuscular immunization	pJHL184:*fliC* ghost can generate significantly high antigen-specific IgY and cell-mediated immune responses and cytokine responses elicited by stimulated splenic T-cells. The elimination of both SE and ST in chicken organs ensures the immunization of the present SE. The ghost vaccine be beneficial in preventing enteric infections in humans.	[65]
*Salmonella enteritidis*	pVAX1-nspA plasmid	Immune cells	Oral immunization	Coadministration of SE ghosts (pVAX1-nspA) and SE ghosts (pVAX1-porB) elicited significant specific humoral and cellular immune responses.	[66]
*Streptococcus suis*	Plain BGs	Immune cells	Subcutaneous immunization	*S.suis* ghosts as candidate vaccine showed the excellent immunogenicity and provided protection against *S.suis* challenge in mice model.	[67]
*Streptococcus iniae*	Plain BGs	Immune cells	Intraperitoneal immunization	Immunization with *S. iniae* ghosts induces immune responses and provides protection against a virulent *S. iniae* challenge.	[68]
*E. coli* O157:EDL 933	pOEVP1 and pOCVP1 plasmids	Immune cells	Intraperitoneal immunization	The VP1 chimeric antigens of BGs are target candidates for a new type of vaccine against hand-foot-and-mouth disease. This vaccine strategy also elicited a stronger immune response against *E. coli* O157:EDL 933.	[69]
*E. coli* O78:K80	pmET32b plasmid	Immune cells	Subcutaneous immunization	The O78:K80 BGs vaccine triggered higher proinflammatory cytokine expression including IL-6, IL-1β and TNFSF15; a higher level of antibody-dependent humoral (IgY and IgA) and cellular immune responses (IFNγ and lymphocyte proliferation).	[70]
*Brucella abortus*	GEM-7Zf+-gntR-SacB-λE	Immune cells	Subcutaneous immunization	The 2308ΔgntR ghost induced high protective immunity in BALB/c mice against challenge with S2308, and elicited an anti-Brucella-specific immunoglobulin G (IgG) response and induced the secretion of interferon gamma (IFN-γ) and interleukin-4 (IL-4). Additionally, 2308ΔgntR ghosts demonstrated strong spleen CD4^+^ and CD8^+^ T cell responses.	[71]
*Salmonella typhimurium*	DENV-EDIII protein	Immune cells	Oral immunization	Significantly elevated titers of EDIII-specific IgG, IgG1 and IgG2a were observed in the immunized mice. Furthermore, lymphocyte proliferative activity and CD3^+^CD4^+^ T-cell subpopulations increased significantly in vitro in re-pulsed splenic T cells compared with those from non-immunized mice.	[72]
*Salmonella enteritidis* (JOL2114)	HA1 protein	Immune cells	Intramuscular & Oral immunization	Protective humoral and cell-mediated immune responses were effectively elicited against both Salmonella and influenza challenge.	[73]
*Neisseria gonorrhoeae*	pVAX1-porB	Immune cells	Oral immunization	Oral immunization with the BGs vaccine candidate elicited greater CD4^+^ and CD8^+^ T cell responses and induced higher IgG responses than *N. gonorrhoeae* DNA vaccine alone.	[74]
*Actinobacillus pleuropneumoniae*	Plain BGs	Immune cells	Intramuscular immunization	A significant systemic increase of IgM, IgA, IgG(Fc’), or IgG(H+L) antibodies reactive with *A. pleuropneumoniae* was measured in GVPs and BVPs.	[75]
*Vibrio cholera*	*V. cholerae* ghosts expressing rVCG-MOMP	Immune cells	Intramuscular immunization	rVCG-MOMP vaccine induced increased local genital mucosal, as well as systemic, Th1 responses. Moreover, T cells from immunized mice could transfer partial protection against *C. trachomatis*.	[76]

## 5. Clinical Trials

A large number of clinical trials for veterinary uses have been conducted (Table 4) using BGs as an adjuvant for immunization against number of infectious diseases, including avian influenza, chicken salmonellosis, *Salmonella typhimurium* immunization and boosting anti-mycobacterial protective immunity [77,78,79]. In addition, BGs have been shown to work as an adjuvant for DNA vaccines, successfully immunizing both mucosal and systemic tissues [34]. Moreover, BGs were conducted for hand-foot-and-mouth disease caused by caused by the Enterovirus genus, such as Enterovirus 71 and the Coxsackie virus [69]. The BG vaccines have been defined in several pathogens, such as *E. coli* O157:H7, Vibrio cholera and *H. pylori* [61,80,81]. The safety and immunogenicity of BG-formulated vaccine have been established in clinical trials with veterinary uses [40].

## 6. Uniqueness of BGs as Delivery System

The BG system is an unusual innovative system for delivery that combines outstanding natural intrinsic properties. The bacterial cell structure was well-studied, revealing many biochemical principles that were subsequently applied to other organisms. Despite the simplicity of the bacterial structure, it has a well-built cell construction which is responsible for many of their unique biological assemblies. As a result of their intrinsic cellular tissue orbit capabilities and ease of manufacture, BGs derived from living bacterial cells have significant advantages as a delivery systems. The uniqueness of BGs as a DDS is the natural constitutes of the cell envelope. The cell envelope contains the plasma membrane and the cell wall. Each has specific structure features that provide particular benefits to the delivery system. BGs represent empty non-denaturated envelopes. The bucket maintains the bioadhesive properties of the natural cell as the ghost preserves the cellular morphology and native surface antigenic structures; these components are responsible for properties like attachment and adhesion, motility and avoiding immune attack. Dissecting bacterial cell envelopes will illustrate details of the main components of both OM and IM help to better understand how the cell wall retains specific properties.

### 6.1. Structural Integrity

Almost every type of bacterial genus has a cell wall, as it is the carbohydrate-containing rigid construction that surroundings the bacterial cell. This exoskeleton provides all prokaryotes several benefits such as the integrity, shape and permeability of the cells. More importantly, it protects the cell from any encircling environments and acts as a filter, allowing selected materials to enter the cells, excluding large and harmful molecules. The core component of the wall is the complexed peptidoglycan molecules that consist of alternating units of N-acetylglucosamine and N-acetylmuramic acid cross-linked by short peptides [56]. The result is a complexed rigid crossed pattern that is very hard and firm, yet porous, allowing movement across the wall.

Bacterial strains are categorized into two groups and differentiated by the thickness of peptidoglycan. Gram-positive bacteria have a multiple peptidoglycan layers forming a thick rigid structure. Spanning this layer is teichoic acid, which is only found in Gram-positive strains. The cell walls of Gram-negative bacteria differ in that they contain a thin peptidoglycan layer covered by an outer lipid layer rich in LPS content [82]. The peptidoglycan layer covers and protects the plasma membrane; the semipermeable phospholipid-bilayer encloses the cytoplasmic content. Sandwiched between the bacterial OM and IM of Gram-negative walls is a periplasmic space, a highly viscus aqueous cellular compartment that is mostly condensed with proteins [83]. The difference in structural between Gram-positive and Gram-negative cell walls allows the optimal selection of bacterial strains for the production of BG delivery system.

The production of the BGs using the E-lysis protein process leads to the fusion of the OM and IM forming a tunnel [84,85]. This process works only on Gram-negative strains. Studies have shown that the total structure of the cell wall is not altered by the E-mediated process. Moreover, the E-specific transmembrane-formed tunnel is not randomly distributed over the cell envelope but is restricted to areas of potential division sites, primarily in the center of the cell or at polar sites [86]. The tunnel sealed by the movement of the C-terminal end of the E-lysis protein from the inner side to the outer side all over the envelope composite straddles the entire pore and fuses the IM and OM at distinct areas [87,88,89].

After expelling the contents of the cytoplasm, the remaining empty sac (ghost) of the bacteria is empty of nucleic acids, ribosomes and other constituents, whereas the IM and OM structures of BGs are well conserved [90]. The well-maintained vessel can be used as a carrier of a variety of medications, antigens and vaccinations. In all BG vaccine applications, regardless the immunization route, addition of adjuvants is not essential. It has been claimed that LPS and peptidoglycan layers, which are portion of the BGs envelope, act as adjuvants. Furthermore, BGs are totally inactivated and therefore incapable of replication. They are DNA-free and consequently do not present a hazard of horizontal gene transmission. What is more, the produced BGs are extremely stable. The stability of BGs against host lysosomes are mainly due to the composition of the OM; the Gram-positive cell wall is almost completely destroyed by lysozymes, yet, with the application of lysozyme to Gram-negative cells, generally the wall is not destroyed to the same extent as in Gram-positive cells [14]. Regarding the stability of BGs, they can be stored at proper temperatures after lyophilization for many years without losing their characteristics [30].

### 6.2. Bioadhesive and Attachments for Targeted Colonization

Various cellular components are related to bacterial cell adhesion and attachment to the host tissues in both pathogenic and non-pathogenic strains of Gram-negative bacteria. As such, the adhesive OM proteins and the components that are used for attachment and movement such as pili, cilia and fimbriae are maintained within BG [91,92,93]. The production process of BGs retains the cellular morphology and envelope sub-component profile of bacteria, while losing some of the functionality of these entities [94,95]. The major component of the OM of Gram-negative bacteria is the LPS, constituting the outermost molecules, which is composed of two biosynthetic units: the lipid A-core and the O-polysaccharide [96,97]. Most biological effects of LPS are related to these two components that play a significant role in the effective colonization of host tissues and adhesion. BGs are nonliving envelopes, in which certain entities are reserved and can play their role in making the BGs an effective drug carrier [98]. Moreover, the presence of a polysaccharide capsule mediates interactions between the bacterium and its direct environment. Moderating capsule expression is a consequence of bacterial growth; conversely, in BGs, this capsule is not present, as a result of killing the bacterial cells where more or less capsule expression will dictate the likely survival of the bacteria in a hostile environment [96,97]. OM vesicles are naturally produced from pathogenic bacterial adhesions and immunomodulatory fusions, and they facilitate bacterial cell-binding and invasion directly. They are effective bacterial virulence factors, as they share in the diverse aspects of the host–pathogen interaction; however, BGs devoid of such virulence as bacterial vesicles are made by active growing cells, not yields of cell lysis or cell death [99,100,101,102].

### 6.3. Immunogenicity

Generally, the immune system is able to exactly distinguish the antigenic biological polymer (glycolipids and/or proteins) and present it to T cells via the major histocompatibility complex proteins. These immune responses (adaptive) have long been considered the ground of antigenic proteins, while polysaccharides are considered T-cell-independent antigens and are not recognized by the adaptive mechanism requiring the action of humoral immunity. Normally, carbohydrates are not recognized by the immune system, thus bacteria have developed polysaccharide capsules, though the latest finding that sugars play a role in immune recognition makes them more attractive targets in the search for antigenic epitopes [103,104,105]. BGs have maintained some antigenic features that are responsible for additional uniqueness as a delivery system.

LPS is the major component in BGs, is known to be toxic and is classified as an endotoxin that elicits a strong immune response when introduced to a host [103,104,105,106]. All BG composites of LPS, including protein-A, O-antigens and peptidoglycan, which are part of the BG envelope complex, act as immunogenicity modulators stimulating humoral as well as cellular immunity [107]. It is assumed that a vaccine that inhibits the microorganism from colonizing the host and that possesses all significant antigenic cell surface factors reflects the most promising way to avoid infection. BGs represent the ideal vaccine candidate for pathogenic bacterial strains, for example enterohemorrhagic *E. coli* (EHEC) strains [64]. BGs also are one of the best vaccine delivery systems, merging targeting of antigen components and offering essential adjuvant activity without the need for further additions. Additionally, BG studies suggest that using BGs as a vaccine candidate effectively stimulate monocytes and macrophages to induce the immune responses. Moreover, stimulating dendritic cells by BGs can be used for active immunization and immunotherapy in situ [108]. As a vaccine candidate and/or carrier BGs, can be used for immunization using various routes: parenteral, oral, buccal and aerosol. Immunization with BGs does not cause clinical side-effects, while providing full protection against clinical disease. All these advantages qualify BGs as a promising carrier and adjuvant for target antigens and vaccinations.

### 6.4. Compartmentalization and Placement of Antigens and/or Medications within BGs

Knowing all about the principles of BGs and structural components will allow their potent and specific application; either BG will be used as a primal form of the empty bucket or it can be recombinantly modified to produce site-specific or directed delivery. BGs are perfectly suited as carriers for target antigens of diverse origin [109]. Similarly, they can be used as delivery vehicles for DNA-Based vaccines [110]. Antigens can be anchored within the IM and OM, in the periplasmic space along with in the inner lumen of BGs. For example, foreign target antigens can be displayed on the surface of BGs as fusion protein with pili or with outer-membrane proteins [109]. Furthermore, BGs can be loaded with active compounds, signifying ideal, target-oriented drug delivery vehicles. Ghosts have a sealed periplasmic compartment and the transfer of proteins into this space massively extends the capacity of BGs or recombinant BGs to function as transporters of foreign antigens, immunomodulators or other medications [109]. Foreign target antigens can be filled within the periplasmic space of BGs or embedded on a recombinant S-layer that filled the periplasm of BGs. S-layer proteins forming shell-like self-assembly structures can be expressed in the selected candidate vaccine strains prior to E-mediated lysis. Foreign epitopes of up to 600 amino acids can be inserted within the flexible surface loop areas of the S-layer, extending the possibilities of ghosts as carriers of foreign epitopes. Moving toward the IM foreign antigens can be anchored to the IM by E′ or L′ anchor sequences or combined E′-L′ anchor sequence or biotinylated streptavidin sequence. Moreover, foreign antigens can be filled into the cytoplasmic bucket of the ghost using recombinant S-layer or any bioactive compounds. Matrices like dextran that are also used to fill the internal lumen of ghosts can be substituted with various ligands to bind the subunit or other materials of interest [30,109,110]. This fact clarifies the higher superiority of BGs when parallel to other inactivated vaccines. Also, there is no foreign antigen size limitation to be implanted, and the capacity of all spaces, including the membranes when used as carriers of foreign antigens and internal lumen of the BGs can be fully utilized.

## 7. Cell, Tissue Uptake and Cellular Inflammatory Response

BG membranes contain a number of microbial-PAMP, including LPS, peptidoglycan or flagella, that are recognized and taken up by various immune (dendritic cells (DCs), macrophages, B and T cells) and non-immune cells (epithelial cells, fibroblasts and keratinocytes). The interaction of these envelope structures with TLR located on the innate immune cells results in the stimulation of stronger adaptive immune responses [111]. Typically, the adjuvant activity of BGs is driven by the interaction of their TLR agonists with either TLR2 or TLR4, resulting in the induction of the innate immune system through the activation and maturation of DCs [112]. The DC-mediated uptake of BG results in the stimulation of proinflammatory cytokines, especially IL-12, which in turn stimulates NK and Th1 cells [113]. Due to the presence of LPS, BGs enhance the antigen-presenting ability of DCs to CD8+ and T-cells, which produces potent cytotoxic cell responses. Additionally, BGs have the ability to trigger the release of cytokine and chemokine in lymphoid and non-lymphoid cells to maximize the recognition of foreign antigens and the development of efficient immune responses [87]. Abtin et al. demonstrated that the endocytosis of flagellated *E. coli* BGs by the non-APCs, keratinocytes, was higher than that of mutated non-flagellated *E. coli* BGs and also with the stronger induction of cytokines [114]. The overall effect of the stimulation of these cell types, along with the release of proinflammatory factors, results in the comprehensive recruitment of innate and adaptive immune cells in addition to the efficient induction, maturation and phagocytic ability of APCs.

## 8. Ideal Drugs to Be Loaded into BGs

BGs possess cellular morphology similar to that of native bacteria, and entire cell surface structures, including OM proteins, adhesions, LPS and the peptidoglycan layer, are conserved [111]. These remarkable bio-recognitive characteristics play a key role in BGs’ adherence to different surfaces of body tissues. Additionally, the BGs’ platform system provides a new promising approach for the delivery of drugs and other biologically active substances. Thus, when it comes to the selection of a drug for loading into/onto BGs to be delivered to a particular site of action, it is very necessary to be taken into consideration that the selected drug is safe to the cell structure of BGs. They are free of any internal content so this lumen can be occupied with interested different drugs as liquid or absorbed to the lipid compartment or specifically anchored to receptors presented in the BGs.

Different studies showed that the lethal action of cytotoxic drugs on the bacterial cell wall requires the active growth of the organisms [115,116]. Since the BGs are nonliving bacterial cell envelopes, in general, it can be said that very little or no effect is expected from the use of drugs that inhibit the synthesis of the bacterial cell wall. As proof of that, *M. haemolytica* ghosts were utilized for the in vitro delivery of the moderate hydrophilic cytostatic drug, doxorubicin (DOX), to human colorectal adenocarcinoma (Caco-2) cells; the improved antiproliferative and cytotoxic activities in the Caco-2 cells were 2 to 3 times more effective compared to the DOX alone [117].

Our recent investigations of the effect of the sparingly water-soluble cytotoxic drug 5-fluorouracil (5-FU) on the cell wall of *E. coli* ghosts revealed that there was no significant impact of 5-FU in 50, 100, 500 and 1000 μg/mL concentrations on the nature and quality of the cell walls of the ghost cells in comparison to untreated ghosts [118]. These findings emphasize what was reported from preceding studies on *E. coli* K12, that 5-FU exerts its lethal action only on living (growing) bacterial cells. In another delivery model, the water-soluble substance calcine was used, and lysis holes were plugged with bacterial membrane vesicles [95]. The polyphenolic compound resveratrol was used to bind unspecifically to the membrane compartments of BGs. An enhanced binding of biotinylated alkaline phosphatase or biotinylated fluorescence-labeled dextrans to a membrane-anchored streptavidin matrix on the IM of BGs showed successful binding within the inner lumen of BGs [21]. Protective and curative effects versus agricultural plant pathogens were reported when *P. cypripedii* ghosts were used as delivery systems for pesticide with the lipophilic fungicide tebuconazole [119].

Under physiological conditions, most bacteria have a net-negative surface charge, making them more likely to adhere to positively charged surfaces [120]. Because of high proportions of phosphatidylserine (negatively charged) on the surface of cancer cells compared to normal cells, a number of amphipathic cationic antimicrobial peptides (CAPs) could be an effective source of cytotoxic agents that could be a potential candidate for BGs delivery [121]. Nevertheless, further investigations are required to discover whether or not CAPs are safe enough for the cell wall of BGs.

Membrane-active agents and surfactants may cause an alteration in the fluidity and/or permeability of the cell membranes, leading to a loss of function [122]. Surfactants and other soluble amphiphiles seem to be bind to a membrane even at very low concentrations, though they may involve subtle alterations in the membrane permeability and membrane lysis and fusion at higher concentrations [122]. As a result, the use of surfactants with BGs should be completely avoided. The use of antimicrobial agents targeting the cell wall might be appropriate for BG delivery unless future studies prove that they have a deleterious effect on BGs’ cell walls.

## 9. Safety Issues of Using BGs

The prevalence of BGs uses in combination with the low manufacturing costs make the BG platform technology a safe and suitable for a targeted DDS, especially for vaccines and active compounds, along with carriers of immobilized enzymes for biotechnology applications. Safety is one of the most important factors for accepting and approving these medical applications. The main source of bacterial virulence is the genetic materials and the typical surface receptors of the BGs, and their living counterparts are being exploited for specific cellular and tissue targeting [109]. For protection causes, researchers inactivated all remaining bacterial DNA in the BG-making process. This may be achieved by the use of staphylococcal nuclease A and/or treatment with β-propiolactone [30]. One of the important BG advantages is that they are dead and do not have a genetic material.

### 9.1. Human Risk of BGs as DNA Vaccine Carriers

Today, a DNA vaccine has been permitted for applications in veterinary field [123]. For human uses, DNA vaccines still need more investigation and developments before use and before they are deemed safe. The FDA still has not approved DNA vaccines using BGs as carriers. This may be due to the necessity of excessive plasmid dosages and little immunogenicity that are most normally attributed to the lack of an effective delivery system [124]. In the last few years, several studies have utilized the BGs as a carrier to deliver DNA vaccines using a simple technique for loading BGs with plasmid DNA [125,126,127,128].

The concept of using BGs prepared from different Gram-negative bacteria as applicant vaccines developed owing to the need for both effective and safe new vaccines. Traditional bacterial delivery systems and viral vaccines with high transaction effectiveness may combine with a risk of return to their original pathogenic hazard. An attenuated bacterial system as DNA complexes and nucleoporation is safer than a viral system due to effective transfection reduction [129]. The new BGs system introduces a new, extremely efficient gene delivery platform as an alternate to existing methods in vaccine development. The safety of BGs is one of the major advantages. A recent in vitro study showed that BGs have no cytotoxic or genotoxic effect on different types of human cells using different BGs species [30]. Moreover, BGs offer a safe, easy to operate and produce an alternate to the traditional antigen bacterial carrier system, with all of the advantages of the later.

### 9.2. Controlling the Risk

Generally, for controlling the risk of the production and loading of BGs with plasmid DNA, researchers use an effective protocol for clear safety. Briefly, self-immobilizing plasmids (pSIP) were introduced as a one step in-vivo and cost-effective procedure. Through this method, the plasmid DNA carrying an operator sequence is bound to a specific DNA binding protein existing in the bacterial IM [130]. In gene therapy, the bacterial backbone sequences and antibiotic-resistant genes are considered a biological safety risk for DNA vaccination and plasmid DNA [30]. To beat this problem, sophisticated versions of pSIP BG-DNA-vaccines, based on minicircle DNA empty of such biologically risky remnants were established. The developed version of pSIP is based on the ParA resolvase system to produce mcDNA, which is bound to the IM receptor.

## 10. Stability Aspects of Using BGs as Delivery Systems

For the purposes of minimizing the undesirable side-effects and maximizing the therapeutic activity, the production of intelligent DDSs is continuously improved [104]. The stability of those intelligent drug delivery systems is one of the biggest challenges that confront pharmaceutical scientists. Despite the robust and well-known immunological advantages of bacterial delivery vectors, there are safety and stability limits with the carrier-based delivery systems [109]. For instance, despite liposomes being promising drug delivery systems, the major disadvantage of liposomal formulation is related to its stability [131,132]. Generally, liposomal chemical instability (hydrolysis and oxidation of lipids) and physical instability (change in size distribution and leakage of entrapped materials) are faced by liposomes upon storage [133]. By contrast, BGs have significant advantages over other carrier-based delivery particles in terms of stability. Owing to the fact that lyophilized BGs remain stable at ambient temperature for several years, they can be kept as lyophilized preparations at 25 °C for long periods without a lack of efficiency [30]. Unlike the currently utilized vaccines that are called chain dependent, BGs-based vaccines can be processed and stored without refrigeration [134].

## 11. Generation vs. Species

BGs are obtained mainly from Gram-negative bacteria, as the lysis process for BGs production works only with Gram-negative bacteria via the fusion of the IM and OM, producing a specific transmembrane-tunnel structure through which all the cytoplasmic content is expelled. On the contrary, Gram-positive bacteria can be killed but not by gene E-lysis due to OM missing [30]. BGs were generated from varied species of different Gram-positive bacteria such as *E. coli* K12 and BL21 strains, *Helicobacter pylori*, *Klebsiella pneumoniae*, *Actinobacillus pleuropneumoniae*, *Erwinia cypripedii*, *Bordetella bronchiseptica*, *Pseudomonas putida*, *Ralstonia eutropha*, *Mannheimia haemolytica*, *Pasteurella multocida*, *Salmonella typhimurium*, *Salmonella enteritidis* and *Vibrio cholera* [135]. Recently, a new protocol “Sponge-like” for BGs preparation was established. By means of applying different chemical compounds to the target bacterial cells in a series of consecutive treatments, BGs were produced from different E. coli strains using the latter Sponge-like protocol and Sponge-like reduced protocol for the preparation of *E. coli* JM109 BGs [136,137].

## 12. Future Prospective

Nowadays, BGs are considered a promising technology platform for different areas: as novel vaccines, as drug carriers for therapeutic approaches in tumor treatment and as novel probiotics. The future of BGs would be based on their great ability to deliver a drug to target sites. However, a number of problems like their immunogenicity, reduced drug concentration available at target sites and the poor internalization of the ghost into cells may be encountered. Subsequently, it is highly important to optimize, validate and authenticate the overall production process of BGs. Simultaneously and for reproducible results, it would also be an urgent need to evaluate the production variables. Safety is one more point of concern associated with bacterial ghosts, as they appear to be inappropriate for immunocompromised patients. Therefore, overall a lot of work is required to be done.

## 13. Conclusions

Targeted DDSs, including biological targeted DDS, offer the enhanced delivery and availability of medication moieties to many different target cells or tissues resulting in a higher efficacy with a better safety profile. BGs represent a unique biological drug carrier for their remarkable features of being non-living cells maintaining all the natural constitutes of the cell envelope. They can be produced either from Gram-negative or Gram-positive bacteria using different approaches like gene E-mediated lysis or Sponge-like protocol, mild high-pressure shock. BGs could be utilized in the biomedical field to serve as drug delivery vehicles, adjuvants or vaccines for the immunogenic strustures avaialable on the surface, protein and antigen carriers, and as a diagnostic tool. BG-based immunization can be achieved to produce significant humoral and cellular immune responses using various routes of administrations without clinical side effects. Since safety is one of the most important factors for accepting and approving BG medical applications and that the main sources of bacterial virulence are the genetic materials and the typical surface receptors, BGs have proved to be safe and suitable for the targeted DDS as they are dead cells and without genetic materials. Moreover, BGs have significant advantages over other carrier-based delivery particles in terms of stability, since lyophilized BGs remain stable at ambient temperature for several years without a lack of efficiency. Unlike the currently marketed vaccines that are cold-chain-dependent, BG-based vaccines can be processed and stored without refrigeration. From the above, it can be concluded that BGs stand as a promising technology platform for different areas, comprised of novel vaccines, drug carriers for therapeutic approaches in tumor treatment and novel probiotics.

## Figures and Tables

**Table 1:** Classification of targeted DDSs and their subdivisions.

Classes	General Classification	Site of Action	Based on Mechanism
Subclasses	1. Active targeting	1. Organ (colonic targeted DDS)	1. Chemical targeted DDS
	2. Passive targeting	2. Therapeutic material (gene carrier)	2. Physical targeted DDS
		3. Cellular uptake (endocytosis, macropinocytosis, and phagocytosis DS)	3. Biological targeted DDS

**Table 4:** Clinical significance/Outcomes of BGs.

Bacterial Ghosts	Disease	Target Cells	Outcomes/Conferred Protection	Developer/ Pharm. Company
*Edwardsiella tarda*	Edwardsiellosis	Fish	*E. tarda* BGs showed a significant systemic and mucosal Ag-specific humoral immune response.	BIRD-C
*Actinobacillus pleuropneumoniae*	Porcine pleuropneumonia	Pig	Ag-specific humoral immune response; increased T helper cytotoxic T cell ratio; complete protection against clinical disease	BIRD-C
*Pasteurella multocida, Mannheimia haemolytica*	Bovine respiratory disease	Cattle	Protective immunity against homologous challenge; cross-reactivity to various Pasteurella serotypes.	BIRD-C
*Salmonella enteritidis*	Salmonellosis/Enteritis and systemic disease	Chicken	Double-immunized chickens showed protection against the intestinal, liver, splenic and ovarian colonization of *S. enteritidis*; Ag-specific lymphocyte proliferative response in immunized chickens.	BIRD-C
*Aeromonas hydrophila*	Hemorrhagic septicemia	Fish	Oral immunization with *A. hydrophila* BGs elicits systemic and mucosal immune responses.	BIRD-C
*E. coli* 0157:H7	EHEC carrier status Diarrhea	Cattle	Induction of EHEC specific antibodies, significant reduction of both duration and total shedding of EHEC offer oral challenge	BIRD-C
*Heamophilus parasuis*	Glässer’s disease	Pig	Piglets immunized with *H. parasuis* BGs exhibited higher levels of T helper cells relevant for protection.	BIRD-C
*Escherichia coli*	Hemorrhagic septicemia	Fish	Ag-specific immune response; protection after challenge (>80%)	BIRD-C
*Bordetella bronchiseptica*	Kennel cough	Dog	BbBG vaccine showed equivalent results when compared to the positive control vaccine (Bronchicine CAe) in terms of safety and efficacy.	BIRD-C
*Flavobacterium columnare*	Columnaris disease	Fish	*Ctenopharyngodon idellus* immunized with *F. columnare* BGs showed a significantly higher Ag-specific immune response.	BIRD-C
*Salmonella typhimurium*	*E. coli* colibacillosis	Pig	Oral immunization of piglets with *S. typhimurium* BGs ETEC fimbriae provides protection to *E. coli* colibacillosis.	BIRD-C
*Salmonella gallinarium*	Fowl typhoid	Chicken	Significant Ag-specific systemic IgG response; increased mRNA level of Th1 cytokines (IFNγ and IL-2).	BIRD-C
*Klebsiella pneumoniae*	Mastitis	Pig	Cross reactivity to related subspecies and clear protection against virulent bacteria	BIRD-C
*Streptococcus iniae*	Streptococcosis	Fish	Tilapia (*Oreochromis niloticus*) immunized with *S. iniae* BGs showed better protection and higher bactericidal activity as compared to formalin-killed vaccines.	BIRD-C

Ag: Antigen; EHEC: Enterohemorrhagic *E. coli*; ETEC: enterotoxigenic *E. coli*; IgG: Immunoglobulin G; IFNγ: interferon gamma; IL-2: interleukin 2; BGs: Bacterial ghosts.

## Data Availability

Not applicable.
